# Distinct cytokine profiles in plasma and tears highlight ophthalmologic inflammation in type 2 diabetes without retinopathy

**DOI:** 10.3389/fmed.2025.1631334

**Published:** 2025-09-15

**Authors:** Rafael Jiménez-López, Laura Martín-Chaves, Ángel Manuel Gutiérrez-García, Ada del Mar Carmona-Segovia, Begoña Mora-Ordoñez, Ana María Sánchez-García, Lourdes Fernández-Romero, Mora Murri, María José Sánchez-Quintero, Germán Berteli-García, Miguel Ángel Sánchez-Chaparro, Vicente Bodí, Jorge Rodríguez-Capitán, Manuel Jiménez-Navarro, Francisco Javier Pavón-Morón, José Lorenzo Romero-Trevejo

**Affiliations:** ^1^Instituto de Investigación Biomédica de Málaga y Plataforma en Nanomedicina (IBIMA Plataforma BIONAND), Málaga, Spain; ^2^Servicio de Urgencias, Hospital Universitario Virgen de la Victoria, Málaga, Spain; ^3^Departamento de Medicina y Dermatología, Facultad de Medicina, Universidad de Málaga, Málaga, Spain; ^4^Servicio de Cardiología y Cirugía Cardiovascular-Área del Corazón, Hospital Universitario Virgen de la Victoria, Málaga, Spain; ^5^Centro de Investigación Biomédica en Red de Enfermedades Cardiovasculares (CIBERCV), Instituto de Salud Carlos III, Madrid, Spain; ^6^Centro de Salud Rincón de la Victoria, Rincón de la Victoria, Spain; ^7^Servicio de Medicina Intensiva, Hospital Universitario Virgen de la Victoria, Málaga, Spain; ^8^Centro de Investigación Biomédica en Red en Fisiopatología de Obesidad y Nutrición (CIBERObn), Instituto de Salud Carlos III, Madrid, Spain; ^9^Servicio de Medicina Interna, Hospital Universitario Virgen de la Victoria, Málaga, Spain; ^10^Servicio de Cardiología, Hospital Clínico Universitario de Valencia, Instituto de Investigación Sanitaria (INCLIVA), Universidad de Valencia, Valencia, Spain; ^11^Servicio de Oftalmología, Hospital Universitario Virgen de la Victoria, Málaga, Spain

**Keywords:** blood, cytokine, diabetes, inflammation, tear

## Abstract

**Introduction:**

Type 2 diabetes mellitus is associated with chronic inflammation and systemic complications, including ophthalmologic manifestations. While blood cytokines serve as inflammatory biomarkers, their expression in tears and correlation with systemic inflammation remain unclear. This study compared cytokine profiles in plasma and tears of well-controlled type 2 diabetes patients and controls, assessing their correlation and potential as biomarkers for disease monitoring.

**Materials and methods:**

This cross-sectional study included 81 participants [40 with type 2 diabetes without retinopathy (T2DM group) and 41 controls (control group)] from primary care centers. Plasma and tear samples were analyzed using a multiplex immunoassay for 27 cytokines. Data were analyzed using ANCOVA (adjusted for age, hypertension, and dyslipidemia), and correlation analyses.

**Results:**

Patients in the T2DM group exhibited distinct inflammatory profiles. Plasma levels of IL-2 (*P* < 0.05), IL-7 (*P* < 0.05), IL-9 (*P* = 0.001), and CCL4 (*P* < 0.01) were significantly lower, while tear levels of IL-6 (*P* < 0.01), CXCL8 (*P* = 0.001), IL-15 (*P* < 0.05), CCL5 (*P* < 0.001), and VEGF (*P* < 0.01) were elevated compared to controls. No significant correlations were observed between plasma and tear cytokines, suggesting independent regulation of systemic and ophthalmologic inflammation. Tear cytokines exhibited stronger intra-fluid correlations than plasma (98.4% vs. 66.5%), with minimal plasma-tear correlations (3.6%). Age influenced most tear cytokines (24/27 analytes) but had a weaker effect on plasma cytokines.

**Conclusion:**

Despite glycemic control, patients with type 2 diabetes exhibited increased tear cytokines in the absence of diagnosed retinopathy, contrasting with reduced plasma cytokines. The lack of correlations suggests localized ophthalmologic inflammation independent of systemic inflammation, highlighting a persistent risk of retinal vascular damage in type 2 diabetes.

## 1 Introduction

Type 2 diabetes mellitus is a chronic metabolic disease with a high global prevalence, and its incidence continues to rise (1). This condition is particularly concerning due to the multiple complications it can cause in various target organs, including the cardiovascular, renal, neurological, and ocular systems (2). In this context, regular ophthalmologic evaluations become essential for diabetic patients, not only to prevent future complications but also as indicators of disease progression. Therefore, fundus examinations are routinely performed as a screening method to detect potential early complications (3).

Research on the role of inflammation in type 2 diabetes has grown significantly, revealing a chronic low-grade inflammatory state linked to insulin resistance, characterized by elevated levels of inflammatory mediators (4). Studies have shown that patients with type 2 diabetes exhibit increased concentrations of C-reactive protein (CRP) and interleukin-6 (IL-6), both of which are associated with a higher risk of diabetes-related complications (5). Furthermore, cytokine imbalances have been identified in diabetic patients, with upregulated pro-inflammatory cytokines such as IL-2, IL-5, IL-18, and tumor necrosis factor-alpha (TNF-α) observed in those with non-proliferative diabetic retinopathy compared to non-diabetic individuals(6). These findings suggest that inflammation plays a key role in the pathophysiology of type 2 diabetes and its complications, highlighting the potential of inflammatory biomarkers in disease monitoring and risk assessment. Since cytokines are present in blood, other biological fluids, and tissues, have emerged as fundamental tools for the early detection of pathological conditions or abnormalities as promising biomarkers (7, 8). For example, the detection of cytokines in tears has been investigated in various ophthalmologic diseases, such as keratoconus (9) and dry eye (10). Additionally, the use of cytokines as potential inflammatory biomarkers has been evaluated in patients with ischemic heart disease (11, 12).

Analyzing inflammation in diabetic patients is crucial not only for understanding disease progression and management but also for identifying potential biomarkers that can aid in monitoring and treatment. Understanding the evolution of inflammation at both systemic and ophthalmologic levels in diabetic patients under treatment could provide valuable insights into diabetes progression and the effectiveness of current therapies (13).

This study aims to compare the inflammatory status of patients with well-controlled type 2 diabetes without retinal damage diagnosis at both systemic and ophthalmologic levels by evaluating a range of cytokines, including interleukins, chemokines, and trophic factors, obtained from plasma and tear samples. By simultaneously analyzing these biomarkers in the same individuals, this study represents the first attempt to directly correlate inflammation in a target organ with systemic inflammation in individuals with type 2 diabetes.

## 2 Material and methods

### 2.1 Patients and eligibility criteria

This cross-sectional study included 108 participants recruited from health centers in Málaga province, Spain, between May and June 2023. After applying inclusion and exclusion criteria, the final sample comprised 81 patients, categorized into the T2DM group (40 patients with well-controlled type 2 diabetes) and the control group (41 non-diabetic individuals).

Inclusion criteria required men and women aged 18 years or older who provided voluntary, informed consent. For the T2DM group, participants were selected from patient lists at family medicine clinics using systematic, non-random sampling (every fourth patient with type 2 diabetes was selected; if a patient did not meet the inclusion criteria, the next individual on the list was chosen) to minimize selection bias. Patients in this group had a diagnosis of type 2 diabetes for more than 2 years and were receiving medical treatment. Controls were non-diabetic individuals from the same clinic lists, selected using identical sampling criteria.

Exclusion criteria included individuals unable to attend the health center, those with a confirmed or suspected active infectious disease at recruitment, a prior diagnosis of diabetic retinopathy or retinal damage, chronic inflammatory diseases (including autoimmune conditions), pregnant or breastfeeding women, individuals undergoing hormonal therapy, and those with cognitive impairments that could impede understanding of the study’s purpose and procedures.

For the T2DM group, absence of diabetic retinopathy was verified through electronic medical record review of the most recent report from the Andalusian population-based diabetic retinopathy screening program (APDR) (14). This program performs systematic retinal photography every 24 months in primary care and refers suspected cases to ophthalmology for confirmation and follow-up. For the purposes of this study, only patients who had undergone screening within the preceding 6 months were eligible, ensuring that retinal status was recently assessed. Individuals with any documented retinal findings consistent with diabetic retinopathy were excluded, as were those lacking a documented screening within this interval. Participants were included only if no referral to ophthalmology follow-up was recorded, thereby confirming the absence of clinically relevant retinopathy.

### 2.2 Clinical evaluations

All participants in the study were scheduled for a primary care consultation and evaluations were conducted by a healthcare team comprising two physicians and one nurse, who attended to all participants.

During the clinical interview, sociodemographic data were collected and confirmed, along with metabolic complications and cardiovascular risk factors, including any history of myocardial infarction and stroke. In addition to these clinical characteristics, information on medications used for cardiometabolic disorders and diabetes was also recorded.

Additionally, eligibility criteria were assessed, and after participants signed the informed consent form, biological samples were collected.

### 2.3 Sample collection and processing

#### 2.3.1 Plasma

Blood samples were collected in the morning after participants had fasted for 8–12 h. Venous blood was drawn into 10 mL K2 EDTA tubes (BD, Franklin Lakes, NJ, United States) and immediately processed to obtain plasma. Specifically, the blood samples were centrifuged at 2,200 × *g* for 15 min at 4 °C, after which the supernatant (plasma) was collected. Plasma samples were then individually characterized, logged, and stored at −80 °C until further analyses. A small aliquot of plasma from each sample was tested for infectious diseases using commercial rapid tests for HIV, hepatitis B, hepatitis C (Strasbourg, Cedex, France), and SARS-CoV-2 (Bio-Connect, Huissen, Netherlands).

#### 2.3.2 Tears

Tear samples were collected from each eye of each patient during the same consultation session as the clinical evaluation and blood collection, using the Schirmer test with Schirmer-Plus^®^ strips (GECIS, Neung sur Beuvron, France). The paper strips were placed in the inferior fornix of each eye without the prior application of topical anesthetic. Samples with less than 6 mm of moisture on the strip after 5 min were excluded. Tear samples were immediately frozen at −80 °C until analysis, as previously reported (12).

For protein elution, each strip was cut into small pieces and incubated overnight at 4 °C in 100 μL PBS containing 0.3% Tween^®^ 20, 0.5% BSA, and a protease inhibitor. Supernatants were collected, and total protein content was quantified by absorbance at 280 nm using a NanoDrop™ One spectrophotometer (Thermo Fischer Scientific, Waltham, MA, USA).

### 2.4 Cytokine determinations

The concentrations of inflammatory mediators in plasma and tear samples were quantified according to the protocol provided by the Bio-Plex Pro™ Human Cytokine 27-plex Assay kit (#M500KCAF0Y; Bio-Rad Laboratories, Hercules, CA, USA). This multiplex panel was selected because it provides a broad and standardized profile of inflammatory mediators relevant to systemic and ocular disease, and it has been successfully applied in our previous studies of cardiovascular and diabetic patients (11, 12).

This immunoassay is based on Luminex^®^ MAGPIX^®^ technology and was conducted at the IBIMA-BIONAND Platform Laboratory at the Málaga Technology Park in collaboration with the University of Málaga. A total of 27 inflammatory mediators were analyzed: interleukin (IL)-1β, IL-1ra, IL-2, IL-4, IL-5, IL-6, IL-7, CXCL8 (IL-8), IL-9, IL-10, IL-12p70, IL-13, IL-15, IL-17A, CCL11 (eotaxin-1), fibroblast growth factor basic (FGF basic), granulocyte colony-stimulating factor (G-CSF), granulocyte-macrophage colony-stimulating factor (GM-CSF), interferon (IFN)-γ, CXCL10 (IFN-γ-induced protein 10, IP-10), CCL2 (monocyte chemoattractant protein, MCP-1), CCL3 (macrophage inflammatory protein-1α, MIP-1α), platelet-derived growth factor (PDGF-BB), CCL4 (MIP-1β), CCL5 (regulated on activation normal T cell expressed and secreted, RANTES), tumor necrosis factor (TNF-α), and vascular endothelial growth factor (VEGF). The 96-well plates were measured using a Bio-Plex MAGPIX™ reader and Bio-Plex Manager™ MP software (Luminex, Austin, TX, USA) in the Proteomics Unit at the Central Research Support Services of the University of Málaga.

All samples were run in duplicate to enhance the reliability and accuracy of measurements and to minimize measurement bias based on previous determinations. For samples with an optical density (OD) lower than the limit of detection in the multiplex assay but higher than the background (zero values), the assignment of concentrations was arranged from the sample with the lowest OD, which was assigned with half of the minimum concentration that could be interpolated in the standard curves (15). The intra-assay coefficient of variability (CV) was less than 7% and the inter-assay CV was less than 8%. Concentrations of these inflammatory mediators were measured in pg/mL or ng/mL.

### 2.5 Statistical analysis

Data were presented as the number and percentage of events [*n* (%)], mean and standard deviation (mean ± SD), or median and interquartile range [median (IQR, 25%−75%)], according to variable type and distribution.

The statistical significance of differences in categorical variables was assessed with Fisher’s exact test, while differences in continuous variables were evaluated using either the Mann-Whitney U test for non-normally distributed variables or the Student’s *t*-test for normally distributed variables. To control the false discovery rate (FDR) arising from multiple comparisons between the T2DM and control groups, the Benjamini-Hochberg procedure was applied to calculate adjusted *P*-values (*Q*-values).

Multiple correlation analyses between plasma and tear cytokine concentrations were conducted using the Spearman correlation coefficient (rho). Tear cytokine concentrations for each patient were calculated as the mean of both eyes, with a mean coefficient of variation <6%. Analysis of covariance (ANCOVA) (F statistic) was used to assess plasma and tear cytokines based on type 2 diabetes diagnosis, adjusting for independent variables and covariates to minimize bias. Raw cytokine concentration data were inverse-transformed to approximate a normal distribution and meet ANCOVA assumptions. Estimated marginal means and 95% confidence intervals (95% CIs) of the inverse-transformed cytokine concentrations were reported.

A *post hoc* power analysis indicated that the final sample size (*n* = 81) achieved approximately 80% power to detect a moderate-to-large effect (*d*≈0.63) at a 5% significance level, which is considered sufficient for identifying differences in cytokine profiles between the T2DM and control groups (16).

All statistical analyses were conducted using GraphPad Prism version 5.04 (GraphPad Software, San Diego, CA, USA) and IBM SPSS Statistics version 22 (IBM, Armonk, NY, USA). *P*-values less than 0.05 were considered statistically significant.

### 2.6 Ethics

Written informed consent was obtained from each participant after a full description of the study for publication of clinical details. All procedures and protocols were approved by the Ethics Committee of the Andalusian Health Service (Portal de Ética de la Investigación Biomédica de Andalucía-PEIBA, Consejería de Salud y Familias, Junta de Andalucía) (code: 0898-N-23), in compliance with the Ethical Principles for Medical Research Involving Human Subjects adopted in the World Medical Association Declaration of Helsinki (64th WMA General Assembly, Fortaleza, Brazil, October 2013) and Recommendation No. R (97) 5 of the Committee of Ministers to Member States on the Protection of Medical Data (1997), and Spanish data protection act [Regulation (EU) 2016/679 of the European Parliament and of the Council 27 April 2016 on the protection of natural persons with regard to the processing of personal data and on the free movement of such data, as well as with Organic Law 3/2018 of December 5, on Personal Data Protection and Guarantee of Digital Rights. All collected information was managed in accordance with FAIR principles and practices, using alphanumeric codes to ensure participant privacy and confidentiality.

## 3 Results

### 3.1 Demographics and clinical characteristics

An overview of baseline demographic and clinical characteristics is provided in Table 1. The total sample (*n* = 81) comprised a balanced distribution of women (49%) and men (51%), with a median age of 64 years and a median BMI of 28.7 kg/m^2^. Participants were predominantly overweight (53%) and showed a high prevalence of hypertension (67%) and dyslipidemia (53%) but a low proportion of smokers (16%).

**TABLE 1:** Baseline demographic and clinical characteristics.

Variable		Group			*P*-value
		Total	Control	T2DM	
		*n* = 81	*n* = 41	*n* = 40	
Sex [*n* (%)]	Female/ Male	41 (50.6) 40 (49.4)	24 (58.5) 17 (41.5)	17 (42.5) 13 (57.5)	0.149 ^a^
Age (years)	Mean ± SD	64.1 ± 9.5	60.9 ± 9.4	67.3 ± 8.5	0.002 ^b^
BMI (kg/m^2^)	Median (IQR)	28.7 (6.0)	27.9 (6.3)	29.0 (6.2)	0.158 ^c^
Obesity ^d^ [*n* (%)]	Normal	9 (11.1)	6 (14.6)	3 (7.5)	0.691 ^c^
	Overweight	43 (53.1)	22 (53.7)	21 (52.5)	
	Class I	19 (23.5)	4 (9.8)	10 (25.0)	
	Class II	10 (12.3)	4 (9.8)	6 (15.0)	
	Class III	0 (0.0)	0 (0.0)	0 (0.0)	
Hypertension [*n* (%)]		54 (66.7)	19 (46.3)	35 (87.5)	< 0.001 ^a^
Dyslipidemia [*n* (%)]		43 (53.1)	15 (36.6)	28 (70.0)	0.003 ^a^
Smoking [*n* (%)]		13 (16.0)	8 (19.5)	5 (12.5)	0.390 ^a^
Acute myocardial infarction [*n* (%)]		5 (6.2)	0 (0.0)	5 (12.5)	0.019 ^a^
Ischemic stroke [*n* (%)]		3 (3.7)	0 (0.0)	3 (7.5)	0.074 ^a^
Glycated hemoglobin (%)	Median (IQR)	5.9 (1.0)	5.7 (0.4)	6.4 (1.1)	< 0.001 ^c^
Medication use (Pharmacological class) [*n* (%)]	Thiazide diuretics	19 (23.5)	3 (3.7)	16 (40.0)	< 0.001^a^
	Loop diuretics	3 (7.3)	0 (0.0)	3 (7.5)	0.241^a^
	ACE inhibitors/ARBs	45 (55.6)	15 (36.6)	30 (75.0)	<0.001^a^
	Calcium channel blockers	7 (8.6)	2 (4.9)	5 (12.5)	0.264^a^
	Fibrates	16 (19.8)	9 (22.0)	7 (17.5)	0.781^a^
	Statins	28 (34.6)	0 (0.0)	28 (70.0)	<0.001^a^
	Ezetimibe	6 (7.4)	0 (0.0)	6 (15.0)	0.012^a^
	Beta-blockers	11 (13.6)	1 (2.4)	10 (25.0)	0.003^a^
	Alpha-blockers	2 (2.5)	1 (2.4)	1 (2.5)	> 0.999^a^
	Antiplatelet agents	14 (17.3)	2 (4.9)	12 (30.0)	0.003^a^

(^a^) *P*-value calculated using the chi-square test or Fisher’s exact test;

(^b^) *P*-value calculated using the Student *t* test;

(^c^) *P*-value calculated using the Mann-Whitney *U* test;

(^d^) Obesity was categorized according to BMI (kg/m^2^) into normal (<18.5), overweight (18.5–24.9), obesity class I (30.0–34.9), obesity class II (35.0–39.9), and obesity class III (≥40.0). ACE, angiotensin-converting enzyme; ARBS, angiotensin II receptor blockers; BMI, body mass index; IQR, interquartile range; SD, standard deviation.

Comparison between the T2DM and control groups revealed significant differences in age (*P* < 0.01), hypertension (*P* < 0.001), and dyslipidemia (*P* < 0.01). Patients with type 2 diabetes had a higher median age (67 years), and a higher prevalence of hypertension (88%) and dyslipidemia (75%) compared to controls. Notably, all cases of acute myocardial infarction (13%) and ischemic stroke (8%) in the sample were observed among patients with type 2 diabetes, with no cases reported in the control group. Furthermore, patients with controlled type 2 diabetes had a median glycated hemoglobin level of 6.4%, which was significantly higher than that of the control group (*P* < 0.001).

Regarding pharmacological treatment, patients with type 2 diabetes showed significantly greater use of cardiometabolic medications. In this group, 75% were prescribed ACE inhibitors or ARBs (*P* < 0.001), 70% statins (*P* < 0.001), 15% ezetimibe (*P* < 0.05), 25% beta-blockers (*P* < 0.01), and 30% antiplatelet agents (*P* < 0.01). No significant differences were observed in the use of diuretics, calcium channel blockers, fibrates, or alpha-blockers.

As shown in Supplementary Table 1, patients with type 2 diabetes had a median disease duration of 9.5 years and were primarily treated with metformin (87.5%) and SGLT2 inhibitors (62.5%).

### 3.2 Cytokines

Inflammatory mediator concentrations were determined from plasma and tear samples of participants to investigate differences between the T2DM and control groups using the Mann-Whitney *U* test.

#### 3.2.1 Plasma concentrations

Significant differences were found between the two groups in only four analytes (Table 2). Specifically, patients with type 2 diabetes showed a significant decrease in IL-9 (*U* = 389, *P* < 0.001) and CCL4 (*U* = 774.5, *P* < 0.001) concentrations compared to control participants. Conversely, a significant increase was observed in CCL11 (*U* = 1081.5, *P* < 0.05) and VEGF (*U* = 1058.5, *P* < 0.05) concentrations. However, after adjustment for multiple comparisons, only IL-9 and CCL4 remained significant.

**TABLE 2:** Plasma cytokine concentrations.

Variable		Group		*P*-value ^a^
		Control	T2DM	
		*n* = 41	*n* = 40	
IL-1β (pg/mL)	Median (IQR)	7.40 (11.36)	10.25 (7.79)	0.443
IL-1ra (ng/mL)	Median (IQR)	1.37 (0.59)	1.34 (0.40)	0.288
IL-2 (pg/mL)	Median (IQR)	18.31 (7.76)	14.43 (7.74)	0.068
IL-4 (pg/mL)	Median (IQR)	21.57 (10.86)	22.14 (13.37)	0.653
IL-5 (pg/mL)	Median (IQR)	190.5 (168.9)	269.9 (153.9)	0.078
IL-6 (pg/mL)	Median (IQR)	8.97 (9.88)	11.17 (8.24)	0.631
IL-7 (pg/mL)	Median (IQR)	55.93 (36.41)	34.98 (41.76)	0.053
CXCL8 (pg/mL)	Median (IQR)	23.62 (12.63)	25.22 (11.97)	0.636
IL-9 (ng/mL)	Median (IQR)	3.18 (0.66)	2.88 (0.60)	**<0.001**
IL-10 (pg/mL)	Median (IQR)	14.68 (10.76)	14.68 (10.76)	0.357
IL-12p70 (pg/mL)	Median (IQR)	35.57 (17.57)	35.57 (17.57)	0.527
IL-13 (pg/mL)	Median (IQR)	13.49 (8,17)	11.43 (11.3)	0.185
IL-15 (pg/mL)	Median (IQR)	903.4 (276.0)	903.4 (276.0)	0.566
IL-17 (pg/mL)	Median (IQR)	30.17 (16.95)	24.52 (16.25)	0.779
CCL11 (pg/mL)	Median (IQR)	426.6 (219.0)	572.2 (349.0)	0.014
FGF basic (pg/mL)	Median (IQR)	114.52 (38.04)	95.29 (19.23)	0.095
G-CSF (pg/mL)	Median (IQR)	128.2 (125.6)	143.9 (125.6)	0.621
GM-CSF (pg/mL)	Median (IQR)	12.95 (7.30)	13.61 (5.28)	0.060
IFN-γ (pg/mL)	Median (IQR)	41.48 (22.05)	41.48 (19.84)	0.801
CXCL10 (ng/mL)	Median (IQR)	1.73 (1.07)	1.47 (1.04)	0.117
CCL2 (pg/mL)	Median (IQR)	108.76 (80.39)	118.57 (52.68)	0.107
CCL3 (pg/mL)	Median (IQR)	7.12 (3.24)	6.19 (3.44)	0.665
PDGF-BB (ng/mL)	Median (IQR)	1.02 (1.19)	0.73 (0.87)	0.098
CCL4 (ng/mL)	Median (IQR)	1.43 (0.23)	1.24 (0.37)	**<0.001**
CCL5 (ng/mL)	Median (IQR)	12.9 (4.97)	13.93 (6.37)	0.865
TNF-α (ng/mL)	Median (IQR)	154.73 (51.44)	154.73 (51.54)	0.422
VEGF (ng/mL)	Median (IQR)	303.7 (180.1)	423.4 (223.4)	0.024

(^a^) *P*-value calculated using the Mann-Whitney *U* test. Significant differences after adjustment for multiple comparisons (FDR) are indicated in bold (Benjamini-Hochberg corrected significance level *Q* = 0.0037).

#### 3.2.2 Tear concentrations

Analysis of tear cytokine concentrations from both eyes revealed significant differences between the two groups across various analytes (Table 3). Unlike the findings in plasma, cytokines altered in patients with type 2 diabetes were elevated compared to controls. Comparisons between the groups revealed significant increases in tear concentrations of IL-1ra (*U* = 4293, *P* = 0.001), IL-6 (*U* = 4387.5, *P* < 0.001), CXCL8 (*U* = 4476, *P* < 0.001), IL-15 (*U* = 4292.5, *P* = 0.001), G-CSF (*U* = 4014, *P* < 0.05), CCL2 (*U* = 4223, *P* < 0.01), CCL5 (*U* = 4653.5, *P* < 0.001), and VEGF (*U* = 4258.5, *P* = 0.001). After adjustment for multiple comparisons, all significant differences remained.

**TABLE 3:** Tear cytokine concentrations.

Variable		Group		*P*-value ^a^
		Control	T2DM	
		*n* = 82	*n* = 80	
IL-1β (pg/mL)	Median (IQR)	1.09 (0.68)	1.26 (0.87)	0.173
IL-1ra (ng/mL)	Median (IQR)	5.87 (5.56)	8.66 (6.66)	**0.001**
IL-2 (pg/mL)	Median (IQR)	0.77 (0.68)	0.87 (0.51)	0.267
IL-4 (pg/mL)	Median (IQR)	4.95 (4.21)	5.44 (5.18)	0.380
IL-5 (pg/mL)	Median (IQR)	51.43 (37.34)	47.03 (43.12)	0.696
IL-6 (pg/mL)	Median (IQR)	1.19 (2,46)	2.57 (4.29)	**<0.001**
IL-7 (pg/mL)	Median (IQR)	6.99 (4.09)	6.91 (4.88)	0.444
CXCL8 (pg/mL)	Median (IQR)	20.41 (36.21)	45.61 (98.52)	**<0.001**
IL-9 (ng/mL)	Median (IQR)	11.35 (6.36)	11.09 (7.87)	0.461
IL-10 (pg/mL)	Median (IQR)	1.25 (1.07)	1.18 (1.39)	0.515
IL-12p70 (pg/mL)	Median (IQR)	1.60 (1.88)	1.51 (1.64)	0.943
IL-13 (pg/mL)	Median (IQR)	0.24 (0.39)	0.24 (0.58)	0.377
IL-15 (pg/mL)	Median (IQR)	45.52 (54.67)	70.69 (58.64)	**0.001**
IL-17 (pg/mL)	Median (IQR)	2.45 (1.58)	2.40 (2.36)	0.903
CCL11 (pg/mL)	Median (IQR)	2.03 (1.51)	2.29 (2.01)	0.472
FGF basic (pg/mL)	Median (IQR)	4.04 (1.89)	3.95 (2.33)	0.752
G-CSF (pg/mL)	Median (IQR)	4.56 (8.66)	7.16 (17.76)	**0.014**
GM-CSF (pg/mL)	Median (IQR)	0.55 (0.53)	0.66 (0.74)	0.307
IFN-γ (pg/mL)	Median (IQR)	211.4 (251.8)	198.3 (239.4)	0.563
CXCL10 (ng/mL)	Median (IQR)	2.83 (2.07)	3.03 (3.28)	0.133
CCL2 (pg/mL)	Median (IQR)	9.40 (9.19)	12.37 (16.69)	**0.002**
CCL3 (pg/mL)	Median (IQR)	0.23 (0.15)	0.25 (0.31)	0.063
PDGF-BB (ng/mL)	Median (IQR)	1.42 (2.43)	2.26 (1.97)	0.053
CCL4 (ng/mL)	Median (IQR)	4.78 (2.86)	4.49 (3.66)	0.435
CCL5 (ng/mL)	Median (IQR)	1.06 (1.76)	1.98 (1.94)	**<0.001**
TNF-α (ng/mL)	Median (IQR)	13.78 (11.44)	15.2 (14.34)	0.433
VEGF (ng/mL)	Median (IQR)	11.08 (14.04)	15.34 (21.44)	**0.001**

(^a^) *P*-value calculated using the Mann-Whitney *U* test. Significant differences after adjustment for multiple comparisons (FDR) are indicated in bold (Benjamini-Hochberg corrected significance level *Q* = 0.0148). Medians were calculated using tear cytokine concentrations from both eyes.

### 3.3 Cytokines adjusted for age and cardiovascular risk factors

**FIGURE 1:**
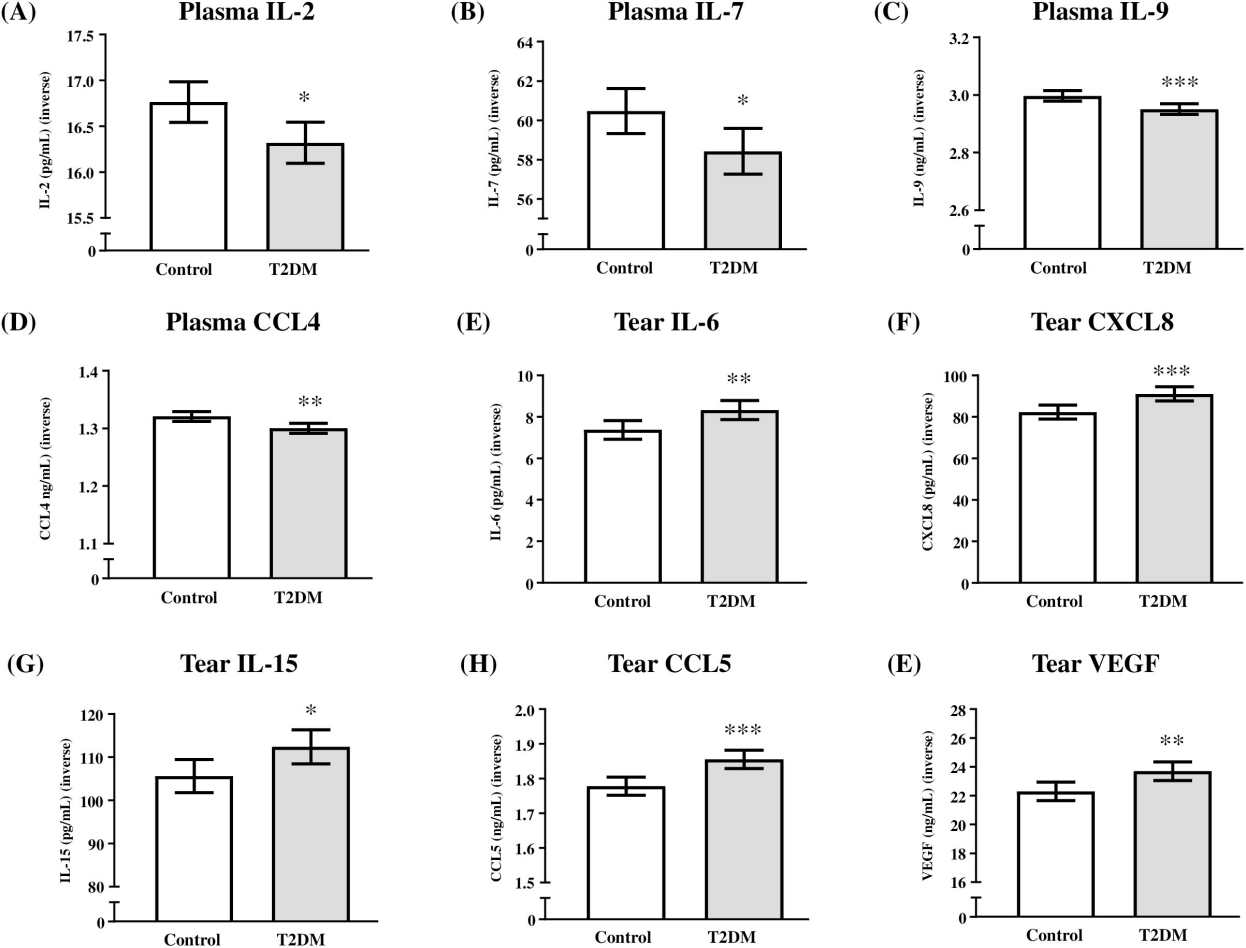
Plasma and tear cytokine levels based on type 2 diabetes diagnosis. **(A)** Inverse-transformed IL-2 concentrations (pg/mL); **(B)** inverse-transformed IL-7 concentrations (pg/mL); **(C)** inverse-transformed IL-9 concentrations (pg/mL); **(D)** inverse-transformed CCL4 concentrations (ng/mL); **(E)** inverse-transformed IL-6 concentrations (pg/mL); **(F)** inverse-transformed CXCL8 concentrations (pg/mL); **(G)** inverse-transformed IL-15 concentrations (pg/mL); **(H)** inverse-transformed CCL5 concentrations (ng/mL); and **(I)** inverse-transformed VGEF concentrations (ng/mL). Bars represent estimated marginal means and 95% CI of inverse-transformed concentrations in the control and T2DM groups. (*) indicates *P* < 0.05, (**) indicates *P* < 0.01, and (***) indicates *P* < 0.001 compared to the control group. Data were analyzed using one-way ANCOVA, with type 2 diabetes diagnosis as the main factor and age, hypertension, and dyslipidemia as covariates. T2DM = Group of patients with type 2 diabetes.

Cytokine concentrations in plasma and tear samples from both eyes were analyzed using ANCOVA, with type 2 diabetes diagnosis as the main factor and age, hypertension, and dyslipidemia as covariates. Given the distribution of analyte concentrations, values were inverse-transformed to meet the assumptions of this parametric statistical procedure. Estimated marginal means and 95% CIs of the inverse-transformed cytokine concentrations are presented in Figure 1.

#### 3.3.1 Plasma levels

Analysis of plasma cytokine levels revealed a significant main effect of type 2 diabetes diagnosis on IL-2 (F(1,81) = 6.83, *P* = 0.011; Figure 1A), IL-7 (F(1,81) = 5.49, *P* = 0.022; Figure 1B), IL-9 (F(1,81) = 11.17, *P* = 0.001; Figure 1C), and CCL4 (F(1,81) = 9.66, *P* = 0.003; Figure 1D). In all cases, type 2 diabetes diagnosis was associated with a significant decrease in the levels of these cytokines in patients with type 2 diabetes compared to controls.

#### 3.3.2 Tear levels

Regarding tear cytokine levels from both eyes, analysis revealed a significant main effect of type 2 diabetes diagnosis on IL-6 (F(1,162) = 7.37, *P* = 0.007; Figure 1E), CXCL8 (F(1,162) = 11.32, *P* = 0.001; Figure 1F), IL-15 (F(1,162) = 5.14, *P* = 0.025; Figure 1G), CCL5 (F(1,162) = 14.73, *P* < 0.001; Figure 1H), and VEGF (F(1,162) = 8.11, *P* = 0.005; Figure 1I). Unlike in plasma, type 2 diabetes diagnosis was associated with a significant increase in the levels of these cytokines in patients with type 2 diabetes compared to controls.

#### 3.3.3 Age and cardiovascular factors on cytokines

Since the statistical analyses were conducted using ANCOVA, with age, hypertension, and dyslipidemia included as covariates, we examined the influence of these covariates on cytokine levels in both fluids. While hypertension and dyslipidemia did not show significant effects on the inverse-transformed cytokine concentrations in plasma or tear samples, age was significantly associated with alterations in various inflammatory mediators, particularly in tear samples.

In plasma, cytokine levels significantly affected by age included IL-1β (F(1,81) = 7.53, *P* = 0.008), IL-9 (F(1,81) = 4.83, *P* = 0.031), CCL11 (F(1,81) = 4.14, *P* = 0.045), CXCL10 (F(1,81) = 6.98, *P* = 0.010), CCL3 (F(1,81) = 4.97, *P* = 0.029), and TNF-α (F(1,81) = 4.29, *P* = 0.042). To further investigate this association with age, we conducted a correlation analysis with the raw concentrations of these analytes. While most of these cytokines exhibited a positive correlation with age, IL-9 showed a significant inverse correlation (rho = −0.35, *P* = 0.001) (Supplementary Table 2).

Notably, analysis of tear cytokine levels revealed that most cytokines (24 out of 27 studied) were significantly affected by age. Significant associations were observed for IL-1β (F(1,162) = 8.08, *P* = 0.005), IL-1ra (F(1,162) = 7.45, *P* = 0.007), IL-2 (F(1,162) = 6.09, *P* = 0.015), IL-4 (F(1,162) = 5.34, *P* = 0.022), IL-5 (F(1,162) = 11.41, *P* = 0.001), IL-6 (F(1,162) = 19.98, *P* < 0.001), CXCL8 (F(1,162) = 13.34, *P* < 0.001), IL-9 (F(1,162) = 6.40, *P* = 0.012), IL-10 (F(1,162) = 4.11, *P* = 0.044), IL-12p70 (F(1,162) = 4.84, *P* = 0.029), IL-13 (F(1,162) = 4.63, *P* = 0.033), IL-15 (F(1,162) = 15.96, *P* < 0.001), IL-17 (F(1,162) = 8.19, *P* = 0.005), CCL11 (F(1,162) = 3.95, *P* = 0.049), FGF basic (F(1,162) = 5.44, *P* = 0.021), G-CSF (F(1,162) = 17.08, *P* < 0.001), GM-CSF (F(1,162) = 6.97, *P* = 0.009), CXCL10 (F(1,162) = 6.66, *P* = 0.011), CCL3 (F(1,162) = 8.91, *P* = 0.003), PDGF-BB (F(1,162) = 11.03, *P* = 0.001), CCL4 (F(1,162) = 12.91, *P* < 0.001), CCL5 (F(1,162) = 18.12, *P* < 0.001), TNF-α (F(1,162) = 5.96, *P* = 0.016), and VEGF (F(1,162) = 14.21, *P* < 0.001). Similar to plasma, correlation analysis using the mean cytokine concentrations from both eyes per participant revealed a positive correlation with age (Supplementary Table 2).

### 3.4 Correlation analysis between cytokines in plasma and tear

After measuring cytokine concentrations in plasma and tear samples from patients with type 2 diabetes and controls, we analyzed correlations among these inflammatory mediators in both fluids using plasma concentrations and the mean tear concentrations from both eyes per participant.

**FIGURE 2:**
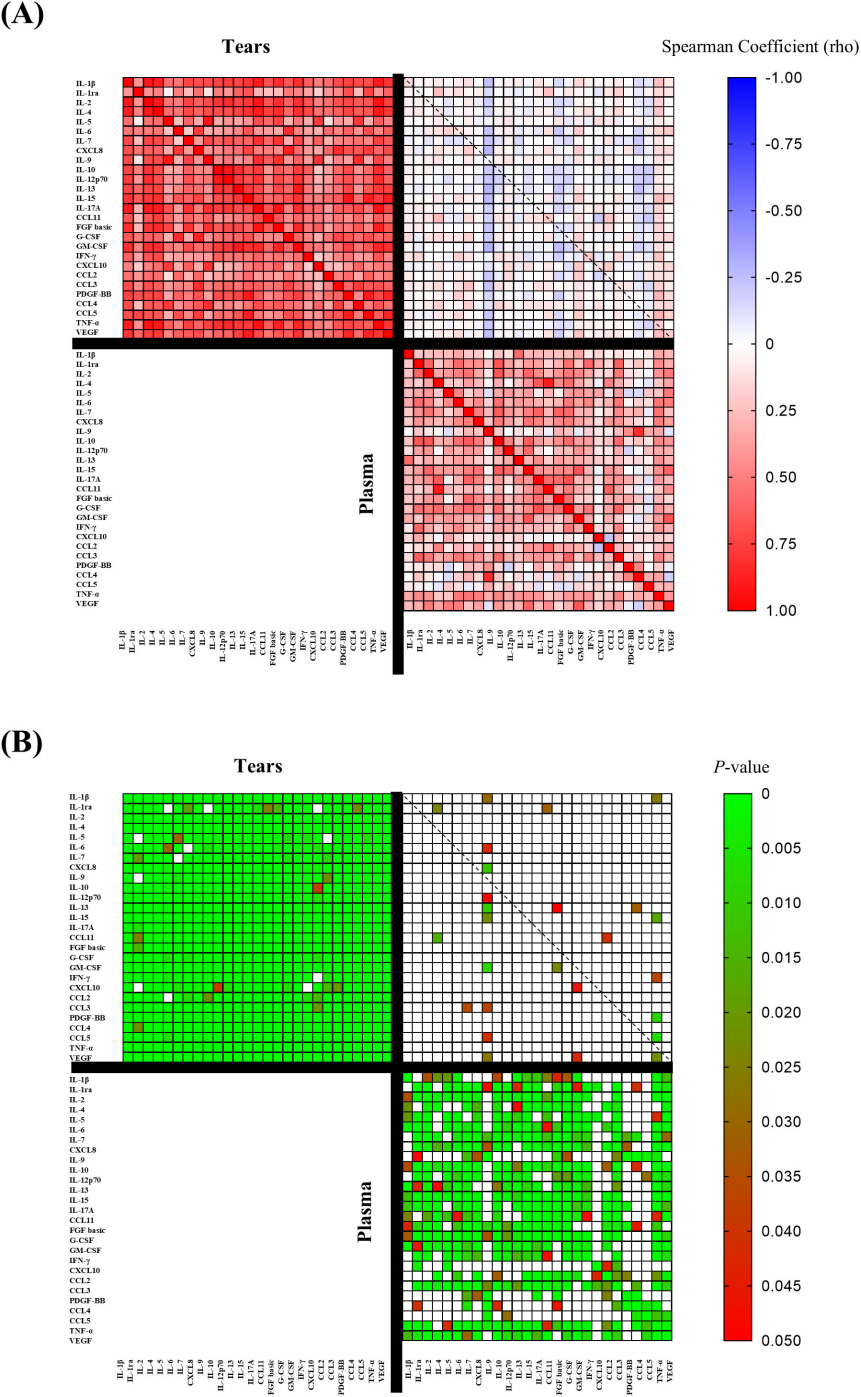
Multiple correlation analysis between plasma cytokine concentrations and the mean tear cytokine concentrations from both eyes per participant in the total sample. **(A)** Spearman correlation coefficients (rho) with a color gradient ranging from rho = + 1.0 (red) to rho = –1.0 (blue) to; and **(B)** Significance of the correlations with a color gradient ranging from *P* < 0.05 (red) to *P* = 0.0 (green). T2DM = Group of patients with type 2 diabetes.

In the total sample, cytokine concentrations in tears showed strong positive correlations, with 98.4% of correlations being statistically significant, whereas plasma cytokines exhibited weaker correlations, with 66.5% reaching significance. Furthermore, only 3.6% of correlations between cytokine concentrations in plasma and tears were significant (Figure 2).

When analyzed separately in the control group (Supplementary Figure 1) and the T2DM group (Supplementary Figure 2), the stronger correlations among cytokine concentrations in tears compared to plasma persisted, as did the lack of association between cytokine concentrations across the two fluids.

Notably, no significant correlations were found between the same inflammatory analyte measured in both plasma and tears, either in the total sample or within each group (Supplementary Table 3). Although an initial analysis suggested a significant positive correlation between IL-1β concentrations in plasma and tears in patients with type 2 diabetes, this association did not remain significant after adjustment for multiple testing (Supplementary Table 3).

Finally, we explored potential associations in the T2DM group between cytokine concentrations in plasma and tears and clinical variables such as HbA1c levels, duration of diabetes, and pharmacological classes of antidiabetic treatment (data not shown). However, no consistent associations were identified, as statistical significance was lost after adjustment for confounding factors.

## 4 Discussion

The increasing prevalence of type 2 diabetes and its association with systemic complications, including ophthalmologic manifestations, underscore the need for biomarkers to aid in disease monitoring and management. Given the central role of inflammation in type 2 diabetes, cytokines have emerged as promising biomarkers for precise disease monitoring (17). This study directly compared cytokine profiles in plasma and tears within the same individuals. Patients with type 2 diabetes exhibited generally reduced plasma cytokine levels compared to controls, whereas tear cytokines were elevated despite no diagnosed retinal damage. Notably, cytokine alterations in plasma and tears did not overlap, and no significant correlations were observed between cytokines in the two fluids. To our knowledge, this is the first simultaneous analysis of blood and tear cytokines in patients with well-controlled type 2 diabetes.

Plasma analysis showed significantly lower levels of IL-2, IL-7, IL-9, and CCL4 in diabetic patients compared to controls. IL-2 and IL-7 are critical immune regulators influencing innate and adaptive immunity (18). IL-2 has been reported to remain elevated following bariatric surgery in patients with diabetes, despite improved glycemic control (19). IL-7, essential for B-cell progenitors, has associations with autoimmune diseases such as rheumatoid arthritis (20). Although CCL4 typically increases in diabetes-related inflammation (21), our study, found reduced levels, possibly due to effective glycemic control. Indeed, CCL4 inhibition has demonstrated benefits in reducing hyperglycemia progression and improving insulin sensitivity (22).

The observed cytokine reductions may reflect effective diabetes management, including pharmacologic treatment. Metformin and acarbose lower IL-2 (23), and insulin reduces CCL4, alleviating beta-cell stress (24). Other antidiabetic drugs, such as liraglutide, suppress oxidative stress and inflammatory responses (25), potentially explaining reduced cytokines in our well-controlled patients. Beyond antidiabetic medications, other widely used treatments in this population may also modulate cytokine levels. Statins, for example, have demonstrated systemic anti-inflammatory actions, including reductions in IL-6, TNF-α, and other proinflammatory cytokines in randomized and animal studies (26). Similarly, ACE inhibitors and angiotensin receptor blockers (ARBs), commonly prescribed for hypertension and nephroprotection in diabetic patients, attenuate inflammatory responses by decreasing cytokine production and enhancing anti-inflammatory pathways (27). In our exploratory analyses, GLP-1 receptor agonists and SGLT2 inhibitors showed statistically significant associations with reduced IL-4 and CCL11 levels. However, these associations were not consistently reproduced when the treatments were considered as independent factors, underscoring the limited statistical power of our cohort and the influence of confounding variables. Taken together, these findings highlight the potential contribution of commonly prescribed medications to the dampened systemic inflammatory profile observed in our patients and support a possible role of modern antidiabetic agents in shaping cytokine responses. This remains an important consideration when interpreting our results in well-controlled clinical settings.

Inflammatory activity in tears is particularly relevant due to ocular complications of diabetes, highlighting the importance of regular ophthalmologic management and fundus examinations (28). While prior studies have focused on tear cytokines in diabetic retinopathy (29, 30), our study identified elevated tear cytokines (IL-6, CXCL8, IL-15, CCL5, and VEGF) in diabetic patients without diagnosed retinopathy, persisting after controlling for age, hypertension, and dyslipidemia. Notably, IL-6 has been linked to diabetes and cardiovascular events, while CXCL8 has been associated with an unfavorable metabolic and lipid profile in type 2 diabetes (31, 32). Even absent significant systemic inflammation, elevated tear cytokines may indicate subclinical microvascular risk, potentially preceding diabetic retinopathy. Chronic localized ocular inflammation can induce vascular dysfunction and neovascularization, hallmarks of retinopathy (7). VEGF, specifically implicated in retinal angiogenesis, strongly predicts proliferative diabetic retinopathy (29). Additionally, elevated IL-6 and CXCL8 similarly promote vascular inflammation and endothelial dysfunction, exacerbating microvascular damage despite systemic inflammation control (31, 32).

Our analysis found no correlations between cytokine levels in tears and plasma, aligning with limited prior research reporting similar independence in uveitis patients (33). This suggests distinct regulatory mechanisms for systemic and ocular inflammation.

IL-1β, crucial in modulating insulin secretion and β-cell apoptosis, increases significantly with diabetic retinopathy severity (34, 35). However, our study found no significant correlation between plasma and tear IL-1β, supporting independent inflammatory pathways between systemic and ophthalmologic responses. An additional consideration is the influence of tear collection methods on cytokine measurements. Schirmer strips, as used in our study, ensure standardized and sufficient sample volumes but may induce reflex tearing and yield higher cytokine concentrations compared to capillary sampling. Systematic reviews and experimental comparisons have emphasized that methodological heterogeneity, particularly related to collection technique, represents a major source of variability across studies. In line with this, recent analyses have shown that Schirmer strips frequently yield higher cytokine concentrations than microcapillary methods, thereby affecting reproducibility and biomarker interpretation (36–38). Importantly, a recent meta-analysis specifically addressing diabetes confirmed that methodological factors substantially contribute to inconsistencies in tear cytokine findings across cohorts (39). Although this limitation cannot be excluded, the consistent elevation of IL-6, CXCL8, CCL5, and VEGF in our patients supports a true local inflammatory signal beyond potential methodological effects.

It should also be considered that other ophthalmologic conditions associated with diabetes might have influenced tear cytokine levels. Although individuals with diagnosed retinopathy were excluded, subclinical retinal changes could still contribute to local inflammatory activity, as reported in early diabetic eye disease (6, 7, 29). In addition, dry eye disease is highly prevalent among patients with type 2 diabetes and has been consistently linked to increased tear concentrations of proinflammatory cytokines such as IL-6, CXCL8, and TNF-α (9, 10). Therefore, part of the cytokine elevation observed in our cohort may reflect concomitant ocular surface inflammation. Nevertheless, the persistence of significant differences after adjusting for age and cardiovascular risk factors supports the interpretation that diabetes itself contributes to a distinctive tear inflammatory profile.

Given that fundoscopy remains the gold standard for diabetic retinopathy screening, tear cytokines could offer a non-invasive alternative for early detection of subclinical ocular inflammation. Longitudinal research is needed to clarify whether elevated tear cytokines predict diabetic eye disease progression, thus guiding personalized ophthalmologic surveillance.

Aging significantly influences cytokine expression, affecting tears through increased inflammatory proteins related to cellular senescence (40, 41). Our findings demonstrated that nearly all tear cytokines were age-dependent, whereas plasma cytokines exhibited fewer age-related changes, reinforcing the concept that tear fluid may be particularly sensitive to aging processes. This differential effect suggests that local ocular tissues may accumulate senescence-associated changes earlier or more prominently than the systemic compartment. Indeed, age-related senescence mechanisms, including the senescence-associated secretory phenotype (SASP), are thought to amplify ocular inflammation by promoting vascular dysfunction, oxidative stress, and impaired tissue repair. More recent reviews have emphasized that aging enhances microvascular susceptibility to metabolic stress, thereby exacerbating diabetes-related retinal and tear film inflammatory alterations (42, 43). In line with these observations, the strong age-dependence of tear cytokines observed in our cohort of patients with type 2 diabetes likely reflects a synergistic interaction between systemic metabolic dysregulation and local age-driven senescence. This interaction could help explain why ocular inflammation emerges even in well-controlled patients without clinically diagnosed retinopathy, suggesting that age not only modulates basal cytokine expression but also amplifies diabetes-related ocular inflammatory responses.

### 4.1 Strengths and limitations

A major strength of this study lies in the direct comparison of cytokine profiles in blood and tears within the same individuals. Glycated hemoglobin (HbA1c), while standard for glycemic control (44), lacks information on underlying inflammation. Our study population comprised well-controlled diabetic patients without retinopathy, minimizing confounding from poor glycemic control.

Several limitations should be acknowledged. The cross-sectional design precludes causality and temporal assessments; longitudinal studies are required to clarify cytokine changes over time. Additionally, a larger, diverse cohort regarding glycemic control and diabetes duration would enhance generalizability. Good glycemic control in our participants might have influenced systemic cytokine profiles, warranting comparative analyses with poorly controlled patients. Potential confounders, including diet, medication, and lifestyle, were not comprehensively controlled. In particular, the strong age-dependence observed in tear cytokine levels represents an additional potential confounder that may amplify ocular inflammation in diabetes; while this limitation was partially addressed by adjusting for age in our analyses, longitudinal studies are needed to disentangle the combined effects of aging and diabetes on tear inflammatory profiles. Finally, the use of Schirmer strips instead of capillary collection may have influenced absolute cytokine concentrations, although this method ensured feasibility and adequate yield for multiplex analysis. In addition, cytokine concentrations were not normalized to total protein content in tears, which may limit direct comparison with studies applying protein-adjusted values, although our approach is consistent with previous reports. Future research should standardize tear collection methods to enhance reproducibility of cytokine-based biomarkers.

### 4.2 Conclusion

Despite limitations, this study demonstrates a distinctive inflammatory tear cytokine profile (elevated IL-6, CXCL8, IL-15, CCL5, VEGF) in type 2 diabetes patients, contrasting with decreased systemic inflammation. The absence of correlations between plasma and tear cytokines indicates ophthalmologic inflammation’s independence from systemic responses. Tear cytokines represent promising non-invasive biomarkers for early detection of ocular inflammatory changes, potentially guiding preventive ophthalmologic care. Future research should validate these findings in broader populations and explore their clinical implications in diabetic eye disease management.

## Data Availability

The datasets presented in this study can be found in online repositories. The names of the repository/repositories and accession number(s) can be found below: https://zenodo.org/records/14846551?token=eyJhbGciOiJIUzUxMiJ9.eyJpZCI6IjUxNjkxMjEwLTE4OWQtNGFkMS04YmU1LTNhMDJlOGQ4ZDRmMiIsImRhdGEiOnt9LCJyYW5kb20iOiJkMjJiYjc xZGNh Mz kzODY3MjhiZTMwZTg5MWFhMTNiMiJ9.7ls650yKUvFHtuwAe2O1lnHlqHOEVwYtPi78PXJfYqP5IbTFUeIjJARvuhFvszZH9eOnaSEvJywu-9ax0KLx6w, doi: 10.5281/zenodo.14846551.
