# Supplementary material for: Distinct cytokine profiles in plasma and tears highlight ophthalmologic inflammation in type 2 diabetes without retinopathy
Source: Front Med (Lausanne). 2025 Sep 15;12:1631334. doi: 10.3389/fmed.2025.1631334 (PMC12477169; doi:10.3389/fmed.2025.1631334)
Supplement: Supplementary file 1 [file Data_Sheet_1.pdf]

Figure S1

(A) Control group (rho values)

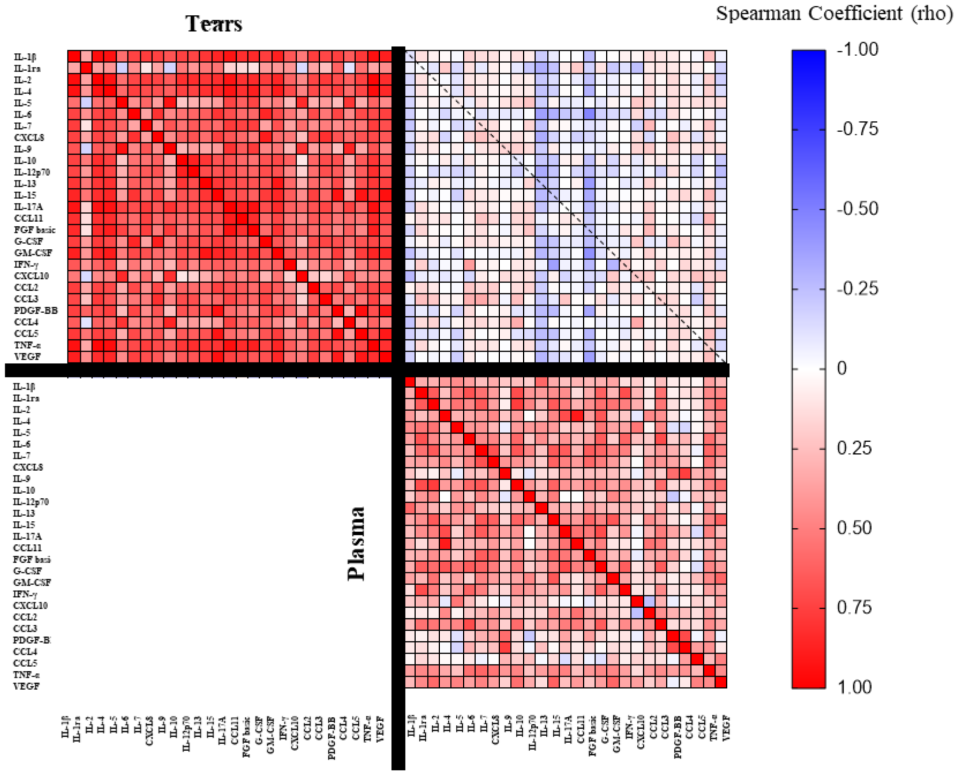

(B) Control group (*P*-values)

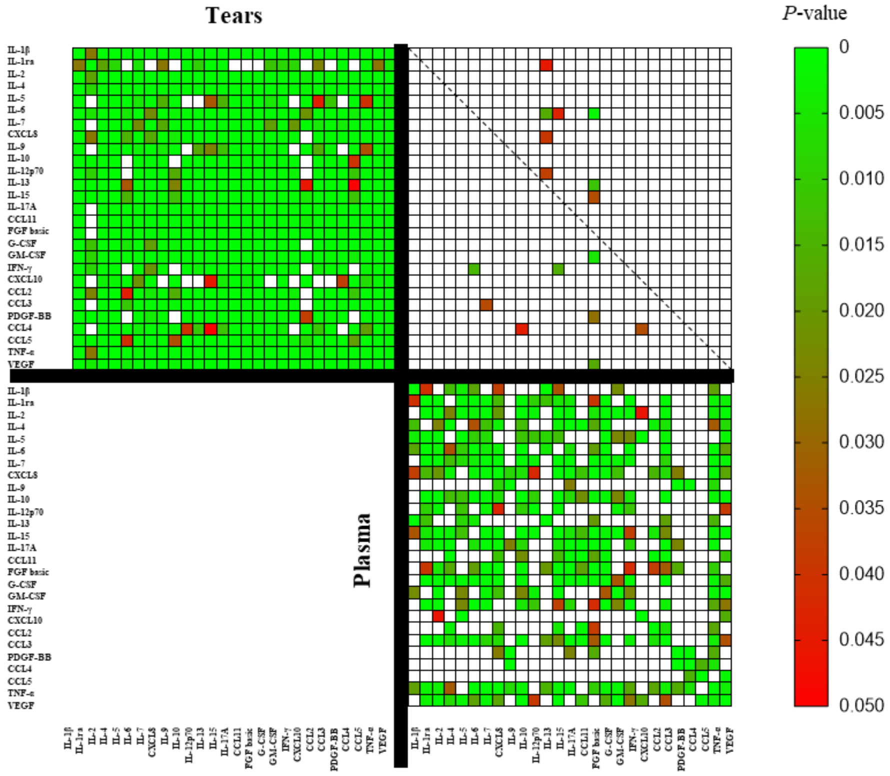

Figure S1. Multiple correlation analysis between plasma cytokine concentrations and the mean tear cytokine concentrations from both eyes per participant in the control group. **(A)** Spearman correlation coefficients (rho) with a color gradient ranging from rho=+1.0 (red) to rho=-1.0 (blue) to; and **(B)** Significance of the correlations with a color gradient ranging from $P < 0.05$ (red) to $P = 0.0$ (green).
