# Supplementary material for: Distinct cytokine profiles in plasma and tears highlight ophthalmologic inflammation in type 2 diabetes without retinopathy
Source: Front Med (Lausanne). 2025 Sep 15;12:1631334. doi: 10.3389/fmed.2025.1631334 (PMC12477169; doi:10.3389/fmed.2025.1631334)
Supplement: Supplementary file 3 [file Table_1.docx]

**Table S1.** Duration of diabetes and antidiabetic medications in the T2DM group.

| **VARIABLE** | | **T2DM** |
| --- | --- | --- |
|  |  | **n = 40** |
| **Duration of diabetes (years)** | Median (IQR) | 9.50 (5.00 - 13.25) |
| **Antidiabetic medication (Pharmacological class)**  **[n (%)]** | Metformin (*biguanide*)  DPP-4 inhibitors  SGLT2 inhibitors  GLP-1 receptor agonists  Insulin | 35 (87.5)  6 (15.0)  25 (62.5)  12 (30.0  9 (22.5) |

Abbreviations: DPP-4 = dipeptidyl peptidase-4; GLP-1 receptor = glucagon-like peptide-1 receptor; IQR = interquartile range; SGLT2 = sodium–glucose cotransporter-2.
