# Supplementary material for: Distinct cytokine profiles in plasma and tears highlight ophthalmologic inflammation in type 2 diabetes without retinopathy
Source: Front Med (Lausanne). 2025 Sep 15;12:1631334. doi: 10.3389/fmed.2025.1631334 (PMC12477169; doi:10.3389/fmed.2025.1631334)
Supplement: Supplementary file 4 [file Table_2.docx]

**Table S2.** Correlation analysis between age and cytokine concentrations in plasma and tear samples for cytokines affected by age in ANCOVA, with type 2 diabetes diagnosis as the main factor.

| **VARIABLE** | **Total** | | | |
| --- | --- | --- | --- | --- |
|  | **Plasma** | | **Tear** | |
|  | **Age (years)** | | **Age (years)** | |
|  | **rho** | ***P*-value ^a^** | **rho** | ***P*-value ^a^** |
| **IL-1β (pg/mL)** | +0.295 | 0.008 | +0.262 | 0.018 |
| **IL-1ra (ng/mL)** | - | - | +0.293 | 0.008 |
| **IL-2 (pg/mL)** | - | - | +0.182 | 0.105 |
| **IL-4 (pg/mL)** | - | - | +0.248 | 0.026 |
| **IL-5 (pg/mL)** | - | - | +0.172 | 0.124 |
| **IL-6 (pg/mL)** | - | - | +0.408 | <0.001 |
| **CXCL8 (pg/mL)** |  |  | +0.387 | <0.001 |
| **IL-9 (ng/mL)** | -0.349 | 0.001 | +0.138 | 0.218 |
| **IL-10 (pg/mL)** | - | - | +0.144 | 0.199 |
| **IL-12p70 (pg/mL)** | - | - | +0.128 | 0.256 |
| **IL-13 (pg/mL)** | - | - | +0.206 | 0.064 |
| **IL-15 (pg/mL)** | - | - | +0.381 | <0.001 |
| **IL-17 (pg/mL)** | - | - | +0.194 | 0.083 |
| **CCL11 (pg/mL)** | +0.314 | 0.004 | +0.226 | 0.042 |
| **FGF basic (pg/mL)** | - | - | +0.187 | 0.094 |
| **G-CSF (pg/mL)** | - | - | +0.388 | <0.001 |
| **GM-CSF (pg/mL)** | - | - | +0.243 | 0.029 |
| **CXCL10 (ng/mL)** | +0.191 | 0.088 | +0.170 | 0.129 |
| **CCL3 (pg/mL)** | +0.217 | 0.052 | +0.240 | 0.031 |
| **PDGF-BB (ng/mL)** | - | - | +0.275 | 0.013 |
| **CCL4 (ng/mL)** | - | - | +0.351 | 0.001 |
| **CCL5 (ng/mL)** | - | - | +0.424 | <0.001 |
| **TNF-α (ng/mL)** | +0.201 | 0.072 | +0.192 | 0.085 |
| **VEGF (ng/mL)** | - | - | +0.347 | 0.001 |

(^a^) *P*-value calculated using the Spearman correlation coefficient. One-way ANCOVA was conducted using type 2 diabetes diagnosis as the main factor, and age, hypertension, and dyslipidemia as covariates. In tears, correlations were calculated using the mean cytokine concentrations from both eyes per participant.
