# Supplementary material for: Distinct cytokine profiles in plasma and tears highlight ophthalmologic inflammation in type 2 diabetes without retinopathy
Source: Front Med (Lausanne). 2025 Sep 15;12:1631334. doi: 10.3389/fmed.2025.1631334 (PMC12477169; doi:10.3389/fmed.2025.1631334)
Supplement: Supplementary file 5 [file Table_3.docx]

**Table S3** Multiple correlation analysis between plasma concentrations and mean tear concentrations from both eyes for the same cytokines.

| **VARIABLE** | **Total** | | **Control** | | **T2DM** | |
| --- | --- | --- | --- | --- | --- | --- |
|  | **n = 81** | | **n = 41** | | **n = 40** | |
|  | **rho** | ***P*-value ^a^** | **rho** | ***P*-value ^a^** | **Rho** | ***P*-value ^a^** |
| **IL-1β (pg/mL)** | +0.144 | 0.199 | -0.113 | 0.481 | +0.427 | 0.006 |
| **IL-1ra (ng/mL)** | +0.016 | 0.888 | +0.018 | 0.910 | +0.112 | 0.493 |
| **IL-2 (pg/mL)** | +0.024 | 0.834 | +0.021 | 0.895 | +0.035 | 0.829 |
| **IL-4 (pg/mL)** | +0.104 | 0.356 | +0.043 | 0.791 | +0.161 | 0.322 |
| **IL-5 (pg/mL)** | +0.003 | 0.975 | +0.058 | 0.719 | +0.001 | 0.996 |
| **IL-6 (pg/mL)** | +0.112 | 0.320 | +0.006 | 0.971 | +0.251 | 0.118 |
| **IL-7 (pg/mL)** | -0.051 | 0.625 | +0.136 | 0.398 | -0.190 | 0.240 |
| **CXCL8 (pg/mL)** | +0.022 | 0.844 | +0.152 | 0.344 | -0.089 | 0.586 |
| **IL-9 (ng/mL)** | -0.044 | 0.694 | +0.158 | 0.323 | -0.590 | 0.070 |
| **IL-10 (pg/mL)** | -0.111 | 0.324 | -0.081 | 0.615 | -0.141 | 0.387 |
| **IL-12p70 (pg/mL)** | -0.054 | 0.634 | -0.033 | 0.837 | -0.069 | 0.672 |
| **IL-13 (pg/mL)** | -0.026 | 0.819 | -0.242 | 0.128 | +0.180 | 0.265 |
| **IL-15 (pg/mL)** | -0.011 | 0.920 | -0.200 | 0.209 | +0.165 | 0.309 |
| **IL-17 (pg/mL)** | +0.057 | 0.613 | -0.002 | 0.989 | +0.111 | 0.497 |
| **CCL11 (pg/mL)** | +0.215 | 0.054 | +0.056 | 0.728 | +0.304 | 0.057 |
| **FGF basic (pg/mL)** | -0.125 | 0.265 | -0.227 | 0.154 | -0.016 | 0.920 |
| **G-CSF (pg/mL)** | -0.037 | 0.743 | -0.091 | 0.571 | +0.086 | 0.599 |
| **GM-CSF (pg/mL)** | +0.131 | 0.245 | +0.032 | 0.845 | +0.213 | 0.187 |
| **IFN-γ (pg/mL)** | +0.210 | 0.059 | +0.199 | 0.213 | +0.196 | 0.226 |
| **CXCL10 (ng/mL)** | +0.116 | 0.301 | +0.202 | 0.204 | +0.129 | 0.463 |
| **CCL2 (pg/mL)** | +0.046 | 0.682 | +0.065 | 0.684 | -0.034 | 0.837 |
| **CCL3 (pg/mL)** | +0.113 | 0.315 | +0.161 | 0.314 | +0.161 | 0.320 |
| **PDGF-BB (ng/mL)** | +0.014 | 0.902 | +0.085 | 0.599 | -0.016 | 0.923 |
| **CCL4 (ng/mL)** | -0.024 | 0.831 | +0.085 | 0.597 | -0.047 | 0.775 |
| **CCL5 (ng/mL)** | +0.086 | 0.445 | +0.004 | 0.979 | +0.096 | 0.554 |
| **TNF-α (ng/mL)** | +0.195 | 0.081 | +0.172 | 0.282 | +0.204 | 0.207 |
| **VEGF (ng/mL)** | +0.177 | 0.114 | -0.028 | 0.863 | +0.324 | 0.041 |

(^a^) *P*-value calculated using the Spearman correlation coefficient. Significant correlations after adjustment for multiple testing (FDR) are indicated in bold (Benjamini-Hochberg corrected significance level *q* = 0.0019).
